# Belief in a COVID-19 Conspiracy Theory as a Predictor of Mental Health and Well-Being of Health Care Workers in Ecuador: Cross-Sectional Survey Study

**DOI:** 10.2196/20737

**Published:** 2020-07-21

**Authors:** Xi Chen, Stephen X Zhang, Asghar Afshar Jahanshahi, Aldo Alvarez-Risco, Huiyang Dai, Jizhen Li, Verónica García Ibarra

**Affiliations:** 1 Business School, University of Nottingham Ningbo China Ningbo China; 2 Faculty of Professions, University of Adelaide Adelaide Australia; 3 CENTRUM Catholica Graduate Business School, Pontifical Universidad Catholica del Peru Lima Peru; 4 Facultad de Ciencias Empresariales y Económicas, Universidad de Lima Lima Peru; 5 School of Economics and Management, Tsinghua University Beijing China; 6 School of Business Administration, Carchi State Polytechnic University Turkan Ecuador

**Keywords:** coronavirus, 2019-nCoV, mental health, psychiatric identification, Latin America, COVID-19, conspiracy, well-being, health care worker, social media, prediction

## Abstract

**Background:**

During the coronavirus disease (COVID-19) pandemic, social media platforms have become active sites for the dissemination of conspiracy theories that provide alternative explanations of the cause of the pandemic, such as secret plots by powerful and malicious groups. However, the association of individuals’ beliefs in conspiracy theories about COVID-19 with mental health and well-being issues has not been investigated. This association creates an assessable channel to identify and provide assistance to people with mental health and well-being issues during the pandemic.

**Objective:**

Our aim was to provide the first evidence that belief in conspiracy theories regarding the COVID-19 pandemic is a predictor of the mental health and well-being of health care workers.

**Methods:**

We conducted a survey of 252 health care workers in Ecuador from April 10 to May 2, 2020. We analyzed the data regarding distress and anxiety caseness with logistic regression and the data regarding life and job satisfaction with linear regression.

**Results:**

Among the 252 sampled health care workers in Ecuador, 61 (24.2%) believed that the virus was developed intentionally in a lab; 82 (32.5%) experienced psychological distress, and 71 (28.2%) had anxiety disorder. Compared to health care workers who were not sure where the virus originated, those who believed the virus was developed intentionally in a lab were more likely to report psychological distress and anxiety disorder and to have lower levels of job satisfaction and life satisfaction.

**Conclusions:**

This paper identifies belief in COVID-19 conspiracy theories as an important predictor of distress, anxiety, and job and life satisfaction among health care workers. This finding will enable mental health services to better target and provide help to mentally vulnerable health care workers during the ongoing COVID-19 pandemic.

## Introduction

During the coronavirus disease (COVID-19) pandemic, social media platforms have become populated with conspiracy theories, which are attempts to explain the ultimate causes of significant social events as secret plots by powerful and malicious groups [1,2]. The most popular examples related to the pandemic include “COVID-19 was developed in a lab,” “people developed COVID-19 to destroy Donald Trump’s presidency,” “COVID-19 is caused by 5G and is a form of radiation poisoning transmitted through radio waves,” and “COVID-19 is Bill Gates’s attempt to take over the medical industry” [3-5]. The latter conspiracy theory alone was mentioned 295,052 times across social media, broadcast media, traditional media, and websites during one week in May 2020 [6]. A national survey in the United Kingdom found that approximately 50% of the population endorsed conspiracy theories to some degree [5].

Individuals’ belief in conspiracy theories has been linked to maladaptive personality traits [7], mental disorders, and lower well-being [8]. However, no research has studied whether a belief in conspiracies about COVID-19 is associated with mental health and well-being. This association is important because posts on social media related to specific COVID-19 conspiracy beliefs are directly assessable; hence, this information is useful to identify people with mental health and well-being issues during the pandemic. In this paper, we explore whether belief in a COVID-19–specific conspiracy theory that the disease was developed intentionally in a lab is a predictor of individuals’ mental health and well-being during the pandemic. In particular, we focus on the mental health and well-being of health care workers, which is a prevalent and emergent issue during the COVID-19 pandemic [9]. The identification of belief in COVID-19 conspiracy theories as a marker of mental health issues in health care workers reveals a new channel for psychiatric screening and health communication [10], opening new avenues of research for medical informatics.

Previous research on COVID-19 has been primarily conducted in the United States, China, and European countries, and there is a need for research in low-and-middle-income countries [11]. This study focuses on Ecuador, where the COVID-19 crisis presents a particularly serious threat for health care workers given the country’s scarce health care resources [12]. We surveyed health care workers in Ecuador from April 10 to May 2, 2020. During this period, there were 26,336 confirmed cases of COVID-19 and 1063 deaths; thus, the small country of Ecuador is among the countries with the highest numbers of cases and deaths per capita in the world [13].

## Methods

### Sample and Procedure

We conducted a web-based survey with a convenience sample that included health care workers in both urban and rural areas. We approached 401 health care workers who worked in hospitals, clinics, emergency response units, medical wards, nursing homes, dental clinics, and pharmacies in the 24 provinces of Ecuador. We received 252 completed surveys (response rate: 62.8%) from 54 health care facilities in 13 provinces (29 facilities in Carchi, 9 facilities in Quito, and 16 facilities from 11 other provinces). Therefore, our sample covered a wide range of provinces in which the severity of the COVID-19 crisis varied.

Ethical approval (20200322) was obtained from Tsinghua University. All participants provided their informed consent, participated voluntarily, and could terminate the survey at any time. The survey was anonymous, and confidentiality of information was ensured.

### Measurements

We assessed the participants’ sociodemographic characteristics, including gender, age, educational level, marriage status, and number of hours of exercise per day during the past week. COVID-19 status was measured by asking “Are you infected with COVID-19?” with answer options of No, Unsure, or Yes. We measured belief in a conspiracy theory specific to COVID-19 by asking participants “From what you’ve seen or heard, what do you think is most likely the origin of the coronavirus?” The four possible responses were 1) It came about naturally; 2) It was developed intentionally in a lab (conspiracy theory belief); 3) It was most likely made accidentally in a lab; 4) I am not sure where the virus originated [14].

We used a brief measure of generalized anxiety disorder, the GAD-7, which has been used broadly to measure anxiety [15]. The GAD-7 consists of seven questions, with a score of 10 or greater indicating generalized anxiety disorder caseness (α=.87). Psychological distress was measured with the 6-item K6 screening scale (α=.90) [16], with a score of 13 representing psychological distress caseness. We conducted logistic regression to analyze the anxiety and psychological distress caseness.

Following the example of previous research [17,18], we used life satisfaction and job satisfaction to measure health care workers’ well-being. Life satisfaction was measured by a satisfaction with life scale containing five items, including “In most ways, my life is close to my ideal” (1=strongly disagree, 7=strongly agree; α=.81) [19]. Job satisfaction was measured with five items, including “I feel fairly satisfied with my present job” (1=strongly disagree, 7=strongly agree; α=.78) [20]. We used linear regression to analyze the participants’ life satisfaction and job satisfaction.

## Results

### Descriptive Findings

Table 1 presents the descriptive findings for the survey responses of the sampled health care workers. Of the 252 health care workers who completed the survey, 61 (24.2%) believed that COVID-19 was developed intentionally in a lab; 52 (20.6%) believed that the virus came about naturally; 35 (13.9%) believed that it was created accidentally in a lab; and the remaining 104 (41.3%) were unsure where it originated.

**Table 1:** Descriptive findings and predictors of health care workers’ mental health and well-being by regression analyses (N=252).

Variable		n (%)	Anxiety		Psychological distress		Life satisfaction		Job satisfaction		
			OR^a^ (95% CI)	*P* value	OR (95% CI)	*P* value	*β* (95% CI)	*P* value	*β* (95% CI)		*P* value
**Belief in the origin of COVID-19^b^**											
	Not sure	104 (41.3)	Reference	N/A^c^	Reference	N/A	Reference	N/A	Reference		N/A
	Developed intentionally	61 (24.2)	4.76 (2.29 to 9.90)	0.000	2.44 (1.20 to 4.98)	0.014	–0.20 (–0.34 to –0.07)	0.004	–0.15 (–0.29 to 0.00)		0.036
	Occurred naturally	52 (20.6)	1.62 (0.73 to 3.59)	0.239	1.08 (0.51 to 2.29)	0.834	0.01 (–0.10 to 0.13)	0.839	0.00 (–0.13 to 0.13)		0.944
	Created accidentally	35 (13.9)	1.12 (0.42 to 3.00)	0.827	0.93 (0.39 to 2.21)	0.877	–0.08 (–0.21 to 0.05)	0.216	–0.09 (–0.23 to 0.06)		0.213
**Marital status**											
	Not married	137 (54.4)	Reference	N/A	Reference	N/A	Reference	N/A	Reference		N/A
	Married	115 (45.6)	1.16 (0.63 to 2.14)	0.636	0.74 (0.41 to 1.32)	0.307	0.15 (0.04 to 0.27)	0.010	0.04 (–0.09 to 0.17)		0.522
**Education**			1.27 (0.91 to 1.76)	0.163	1.24 (0.89 to 1.71)	0.202	0.12 (–0.01 to 0.24)	0.076	0.04 (–0.10 to 0.19)		0.533
	High school	11 (4.4)									
	Technical	9 (3.6)									
	Undergraduate	159 (63.1)									
	Master	43 (17.1)									
	Specialty	30 (11.9)									
**Age (years)**			0.98 (0.94 to 1.01)	0.237	0.97 (0.94 to 1.01)	0.127	0.09 (–0.06 to 0.25)	0.233	0.21 (0.05 to 0.36)		0.006
	18-24	26 (10.3)									
	25-34	125 (49.6)									
	35-44	61 (24.2)									
	45-54	32 (12.7)									
	55-69	8 (3.2)									
**Gender**											
	Female	165 (65.5)	Reference	N/A	Reference	N/A	Reference	N/A	Reference		N/A
	Male	87 (34.5)	1.44 (0.78 to 2.65)	0.244	0.96 (0.55 to 1.70)	0.897	0.10 (–0.02 to 0.22)	0.089	0.02 (–0.11 to 0.15)		0.751
**Daily hours of exercise in the previous week**			0.84 (0.69 to 1.01)	0.069	0.91 (0.77 to 1.07)	0.234	0.15 (0.04 to 0.26)	0.009	0.11 (–0.02 to 0.24)		0.075
	0	90 (35.7)									
	1	78 (31.0)									
	2	27 (10.7)									
	3	24 (9.5)									
	4	8 (3.2)									
	5	10 (4.0)									
	≥6	15 (6.0)									
**Infected with COVID-19**											
	Unsure	70 (27.8)	Reference	N/A	Reference	N/A	Reference	N/A	Reference		N/A
	No	181 (71.8)	0.60 (0.31 to 1.31)	0.113	0.60 (0.33 to 1.12)	0.110	0.14 (0.03 to 0.26)	0.016	0.11 (–0.03 to 0.25)		0.096
	Yes	1 (0.4)	N/A	N/A	N/A	N/A	–0.02 (–0.04 to 0.00)	0.084	–0.06 (–0.09 to –0.03)		0.000

^a^OR: odds ratio.

^b^COVID-19: coronavirus disease.

^c^N/A: not applicable.

### Predictors of Health Care Workers’ Mental Health

As presented in Table 1 and further illustrated in Multimedia Appendix 1, health care workers who believed that the virus was developed intentionally in a lab were more likely to experience psychological distress than those who were unsure of the origin of the virus. The Wald test showed that these health care workers were also more likely to experience psychological distress than those who believed the virus was created accidentally (*χ*^2^_1_=4.24, *P*=.039).

Health care workers who believed that the virus was developed intentionally in a lab were more likely to have anxiety disorder than those who were unsure how the virus originated. The Wald test showed that these health care workers were also more likely to have anxiety disorder than those who believed the virus came about naturally (*χ*^2^_1_=6.42, *P*=.011) and those who believed the virus was made accidentally (*χ*^2^_1_=8.11, *P*=.004).

### Predictors of Health Care Workers’ Well-Being

Health care workers who were married or who exercised for more hours in the previous week reported higher life satisfaction. Those who were not affected by COVID-19 were more satisfied with life than those who were unsure. Health care workers who believed the virus was developed intentionally in a lab had lower life satisfaction than those who were unsure how the virus originated. The Wald test showed that the life satisfaction of these health care workers was also lower than that of health care workers who believed the virus came about naturally (*χ*^2^_1_=7.80, *P*=.006).

Older health care workers had higher job satisfaction. Health care workers who believed that the virus was developed intentionally in a lab had lower job satisfaction than those who were unsure how the virus originated.

## Discussion

### Principal Findings

First, this study revealed that health care professionals can believe in conspiracy theories (61/252 in this sample, 24.2%). A prevalent belief in a conspiracy theory is related to high anxiety and distress of health care workers in Ecuador. Almost one-third (n=82, 32.5%) of the 252 health care workers passed the cutoff for psychological distress, and 71 (28.2%) had anxiety disorder. The proportion of psychologically distressed health care workers in Ecuador was significantly higher than that of health care workers in Iran surveyed from February 28 to 30, 2020 (20.1%, N=304) [21]. The prevalence of anxiety disorder was similar to that in a sample of 5062 health care workers (24.1%) in Wuhan, China, from February 8 to 10, 2020 [22], and higher than that in a sample of 4872 individuals (22.6%) in China surveyed from January 31 to February 2, 2020 [23].

In this study, we found that belief in a conspiracy theory regarding the origin of COVID-19 was associated with lower mental health, life satisfaction, and job satisfaction of health care workers. From a health informatics perspective, belief in a COVID-19–related conspiracy theory provides a marker to identify mentally vulnerable people, who may browse, search, follow, like, discuss, and disseminate COVID-19–related conspiracy theories via social media and other channels. This information can serve as a risk factor to identify individuals who are more susceptible to mental disorders through psychiatric screening via social media [24] at a time when psychological screening, diagnosis, and intervention are rapidly becoming web-based [25].

In addition, this study has important implications for the dissemination of scientific and health information. Previous research has recognized the important role of web-based scientific communication in combating conspiracy theories [1,26]. This study suggests that such communication should acknowledge recipients’ psychological states, such as anxiety and distress, while introducing scientific hypotheses about the origin of the virus [27]. Given that people who believe in conspiracy theories tend to form clusters [4], followers of COVID-19–related conspiracy theories also provide targeted groups for scientific communication and dissemination of mental health information [10].

Finally, belief in the conspiracy theory that COVID-19 was developed intentionally in a lab was associated with reduced job satisfaction of health care workers. Given that the mental health of health care workers is important to sustain their employment and job performance [28], this study highlights the important role of conspiracy theories in assessing the mental health of health care workers, which has profound implications for their overall performance. This is especially important in settings where health care resources are already constrained, such as the COVID-19 pandemic.

### Limitations and Future Research

This study has several limitations. First, the cross-sectional design limits our ability to make causal arguments about the relationship between belief in conspiracy theories and mental health. In future research, experimental designs should be adopted to establish a causal relationship between conspiracy theory belief and mental health. Second, we only focused on health care workers, whose role is especially important during the ongoing COVID-19 pandemic in Ecuador. It is worth investigating whether the effects of belief in conspiracy theories generalize to the general population. Finally, Ecuador is a country that has been severely affected by the pandemic. The extent to which these findings are generalizable to other countries, which face different degrees of threat from the pandemic, remains to be determined. For instance, it may be interesting to investigate whether belief in conspiracy theories about COVID-19 predicts mental health in countries where the social and political systems are severely threatened by the pandemic, because system identity threat is an important cause of adoption of conspiracy theories [29].

### Conclusion

This study provides the first empirical evidence that belief in COVID-19–related conspiracy theories is associated with the mental health and well-being of health care workers. Hence, belief in COVID-19–related conspiracy theories expressed on social media and in interest groups may help identify mentally vulnerable people to enable more targeted identification and communication from a health informatics perspective.

## Appendix

Multimedia Appendix 1 Predicted values and 95% CIs of health care workers’ anxiety (GAD-7 score≥10), distress (K6 score≥13), life satisfaction, and job satisfaction.

## Abbreviations

COVID-19: coronavirus disease
