# Supplementary figures and images for: Belief in a COVID-19 Conspiracy Theory as a Predictor of Mental Health and Well-Being of Health Care Workers in Ecuador: Cross-Sectional Survey Study
Source: JMIR Public Health Surveill. 2020 Jul 21;6(3):e20737. doi: 10.2196/20737 (PMC7375774; doi:10.2196/20737)

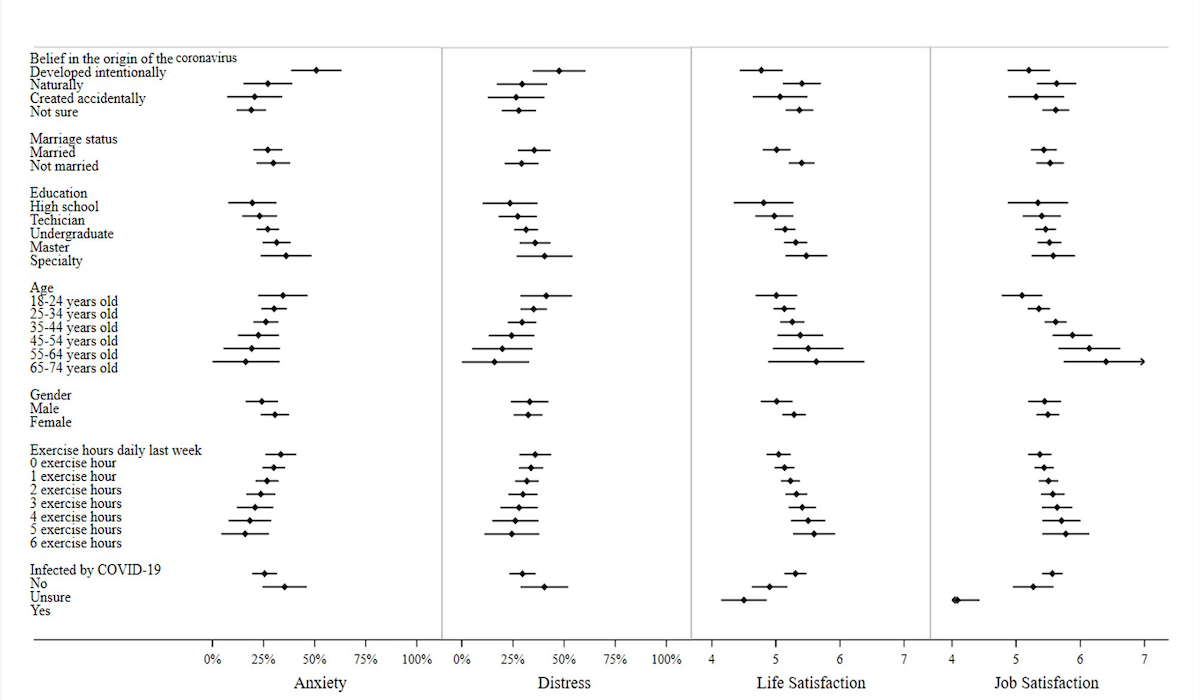

Supplement: Multimedia Appendix 1 [file publichealth_v6i3e20737_app1.png]
